# Effects of first intermediate host density, host size and salinity on trematode infections in mussels of the south-western Baltic Sea

**DOI:** 10.1017/S0031182020002188

**Published:** 2021-04

**Authors:** Claudia Bommarito, David W. Thieltges, Christian Pansch, Francisco R. Barboza, Fabio Pranovi, Martin Wahl

**Affiliations:** 1 Department of Marine Ecology, GEOMAR Helmholtz Centre for Ocean Research Kiel, Hohenbergstr. 2, 24105, Kiel, Germany; 2 Department of Environmental Sciences, University Ca’ Foscari of Venice, Informatics and Statistics, Via Torino 155, 30172, Venice, Italy; 3 Department of Coastal Systems, NIOZ Royal Netherlands Institute for Sea Research, P.O. Box 59, 1790, AB Den Burg Texel, The Netherlands; 4 Environmental and Marine Biology, Åbo Akademi University, Artillerigatan 6, 20520, Åbo, Finland

**Keywords:** Abundance, density, *Mytilus*, prevalence, salinity, size, trematode

## Abstract

Trematode prevalence and abundance in hosts are known to be affected by biotic drivers as well as by abiotic drivers. In this study, we used the unique salinity gradient found in the south-western Baltic Sea to: (i) investigate patterns of trematode infections in the first intermediate host, the periwinkle *Littorina littorea* and in the downstream host, the mussel *Mytilus edulis*, along a regional salinity gradient (from 13 to 22) and (ii) evaluate the effects of first intermediate host (periwinkle) density, host size and salinity on trematode infections in mussels. Two species dominated the trematode community, *Renicola roscovita* and *Himasthla elongata*. Salinity, mussel size and density of infected periwinkles were significantly correlated with *R. roscovita*, and salinity and density correlated with *H. elongata* abundance. These results suggest that salinity, first intermediate host density and host size play an important role in determining infection levels in mussels, with salinity being the main major driver. Under expected global change scenarios, the predicted freshening of the Baltic Sea might lead to reduced trematode transmission, which may be further enhanced by a potential decrease in periwinkle density and mussel size.

## Introduction

Spatial heterogeneity in parasite infection levels in hosts is driven by a wide variety of environmental (both abiotic and biotic) conditions (Anderson and Sukhdeo, 2010). Among the biotic conditions, a major role is played by the distribution and abundance of the various hosts of a parasite's life cycle (Poulin, 1998; Marcogliese, 2001; Morley and Lewis, 2004). This is especially important for parasites with complex life cycles such as trematodes (Poulin and Mouritsen, 2003; Fredensborg *et al*., 2006). In vertebrates, which are the final hosts for trematodes, the adult parasites produce eggs through sexual reproduction. Eggs are then released into the environment and develop into the miracidium stage, which will infect a first intermediate host (most often being a mollusc). In the first intermediate host, sporocysts or rediae will asexually reproduce and develop to cercariae, which are released into the environment and penetrate an invertebrate or vertebrate species as second intermediate host. In these hosts cercariae encyst as metacercariae, waiting to be ingested by the final host. Spatial heterogeneity of trematode infections can therefore be mediated by the abundance and infection status of the various host species involved in the complex life cycle. At a particular life cycle stage, the density and infection levels in the first intermediate host (i.e. the preceding host species in a parasite's life cycle) largely determine the infection levels in the next host (Hechinger and Lafferty, 2005; Thieltges, 2007; Thieltges and Reise, 2007; Galaktionov *et al*., 2015). To date, most of the available studies focused on the density of the final host driving infection levels in downstream first intermediate hosts, whereas effects of the first intermediate host density and infection levels on second intermediate hosts have been much less studied (but see Thieltges, 2007 and Thieltges and Reise, 2007). Apart from these biotic drivers acting at the population level of a host species, infection levels can further differ at the individual host level as conspecifics may differ in their exposure or susceptibility to parasite infections, depending on habitat use, diet and other host traits (Carney and Dick, 1999). Of these host traits, body size has been described as a major driver of infection levels in hosts, with larger hosts offering more space and habitat for parasites, and therefore accumulating more parasites over their lifetime than smaller and younger host individuals (de Montaudouin *et al*., 2000; Mouritsen *et al*., 2003; Thieltges and Reise, 2007; Goedknegt *et al*., 2019).

Heterogeneity in infection levels is also caused by abiotic factors and strong environmental gradients. Temperature, salinity, pH and light are all known to affect the transmission success of free-living stages of many trematode species and thus infection levels in their intermediate hosts (Pietrock and Marcogliese, 2003). In marine ecosystems, salinity plays a particularly important role, and experimental studies on trematode cercariae under laboratory conditions indicate that salinity affects various steps in the transmission from one host to the next (Koprivnikar and Poulin, 2009 and Koprivnikar *et al.* 2010; Lei and Poulin, 2011; Studer and Poulin, 2012; Bommarito *et al*., 2020). Emergence, survival and activity of cercariae, all generally decrease with a reduction in salinity (but see Koprivnikar and Poulin, 2009). Susceptibility of the target host also appears to be negatively affected by reduced salinity (Bommarito *et al*., 2020), further decreasing the chances of transmission to the target host. Hence, based on the information provided by laboratory studies, decreasing salinity is expected to limit the transmission success of trematode parasites, resulting in lower prevalence and abundances in first intermediate hosts at lower salinities. However, little is known on whether this prediction also holds true under field conditions as the majority of the existing field studies on salinity effects have focussed on trematode diversity (Schmidt *et al*., 2003; Thieltges *et al*., 2010; Blanar *et al*., 2011) and rarely examined prevalence and abundance patterns (but see Magalhães *et al*., 2018).

In this study, we investigated the effects of first intermediate host density and salinity on trematode infection levels in mussel second intermediate hosts in the south-western Baltic Sea. The semi-enclosed Baltic Sea basin is characterized by a salinity gradient decreasing from the south-west to the north-east (Snoeijs-Leijonmalm *et al*., 2017). Salinity drops from over 30 in the Skagerrak and Kattegat region to about 2 in the Gulf of Bothnia, representing one of the main drivers of benthic community composition (Zettler *et al*., 2014). Given this unique feature, the Baltic Sea is a suitable region for field studies on the environmental effects on parasitism. Our focus host was the blue mussel (*Mytilus edulis*), which serves as a second intermediate host for trematodes (*Renicola roscovita* and *Himasthla elongata*), as well as a host for other commensals or parasites such as turbellaria (*Paravortex cardii*) or copepods (Dos Santos and Coimbra, 1995; Kaprivin, 2012). For trematodes that infect mussels, periwinkles (*Littorina littorea*) serve as first intermediate, and birds as final hosts (Werding, 1969). By sampling periwinkles and mussels from six locations along a salinity gradient from 22 to 13, we: (i) investigated patterns of parasite infections in periwinkles and mussels and (ii) quantified the effects of first intermediate periwinkle host density and salinity as well as host size on trematode infections in mussels.

## Methodology

### Sampling area and sample collection

**Fig. 1.**
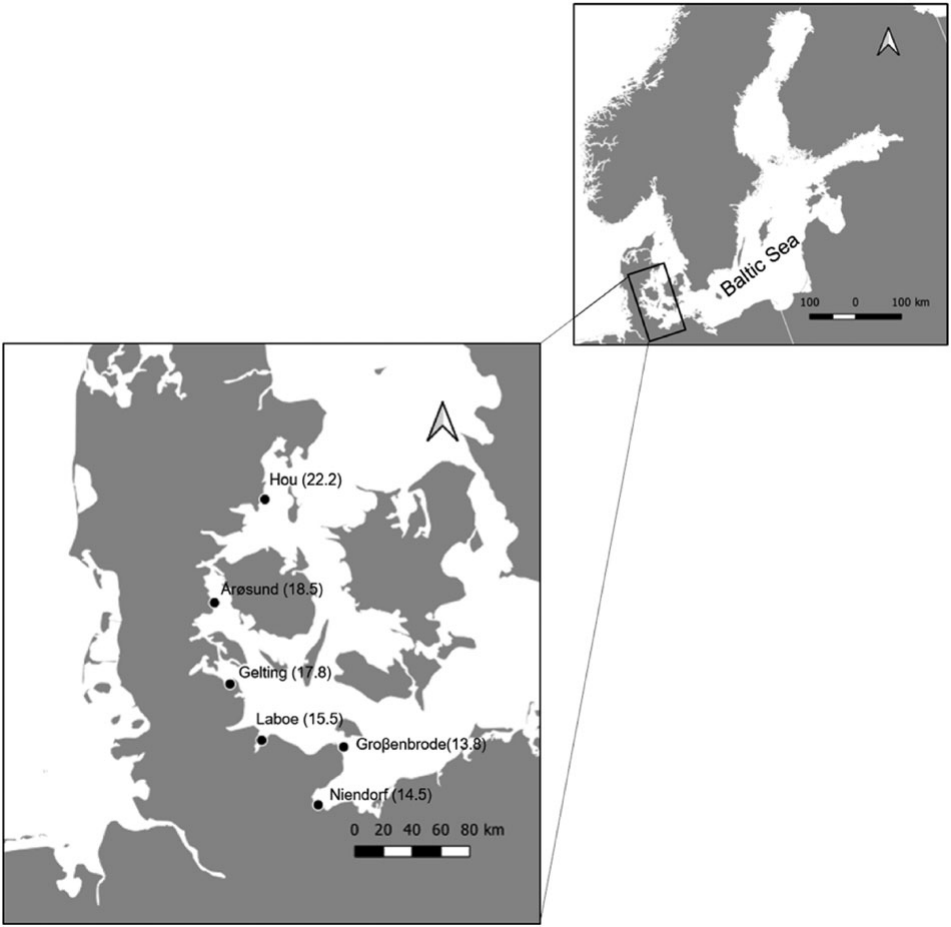
Map of the six sampling locations in Denmark and Germany, with the respective salinities.

A large-scale sampling was performed in June and July 2017 along the south-western coastline of the Baltic (Fig. 1), area that features the steepest salinity slope of the entire Baltic Sea. Six stations were sampled along Danish to Germany coast (Fig. 1). The stations were selected based on geographical distribution and logistical feasibility. Locations below a salinity of 13 were not sampled as the presence of periwinkles drastically decreases (author pers. observ.; Lauckner, 1984; Bonsdorff, 2006). Above a salinity of 22, occurring in the northern region of Denmark, the coast is predominantly characterized by sandy substrate, with a much reduced *L. littorea* abundance that does not allow testing the interactions of both, first and second parasite hosts (author pers. observ.; Eschweiler and Buschbaum, 2011).

At each station, samples were collected in three replicate 50 × 50 cm plots, ~100 m apart from one another. Periwinkle density per plot was categorized in three density classes: low = 0–30; medium = 30–50 and high 50–100 (expressed in individuals per 50 × 50 cm plot). A total of 25–35 periwinkles and 25–30 blue mussels were collected at each station (Table 1). Only specimens larger than 17 mm for periwinkles and 30 mm for mussels were collected, as these sizes are known to be normally infected by common trematodes (Mouritsen *et al*., 1999; Goedknegt *et al*., 2017). Samples were transferred to the GEOMAR Helmholtz Centre for Ocean Research Kiel laboratory. Shell length was measured, and the tissue observed between two glass slides (compressors). Parasites were determined referring to previous descriptions (Werding, 1969; Zander, 1998; de Montaudouin *et al*., 2009) and counted. When periwinkles were infected, almost all tissue area was infested with cercariae and sporocysts or rediae, and accurate abundance estimation was not possible.

**Table 1.** Environmental and biological features of the sampled stations

Station	Salinity (psu)	Temperature (°C)	Density of periwinkle infected with *Renicola roscovita* (no. per 50 × 50 cm)	Density of periwinkle infected with *Himasthla elongate* (no. per 50 × 50 cm)	Number of periwinkles sampled	Number of mussels sampled	Mean mussel length (mm) ± s.e.
Hou	22.2	16.5	5	12.5	30	30	47 ± 1.4
Årøsund	18.5	16.7	20	27.5	30	30	47 ± 1.3
Gelting	17.8	17.0	2.5	0	35	25	49 ± 2.1
Laboe	16.5	17.7	0	2	35	30	43 ± 1.0
Niendorf	14.5	17.8	0	0	25	30	41 ± 1.1
Großenbrode	13.8	17.7	0	0	30	25	40 ± 1.0
Total					220	195	

Salinity is reported based on annual mean of the year 2017, and temperature is based on the mean of the summer months June, July and August of 2017 (Copernicus dataset, see ‘Methodology’ section in the text).

Salinity and temperature data for all sampling locations were retrieved from the Baltic Sea physics reanalysis product provided by Copernicus Marine Environment Monitoring Service (http://marine.copernicus.eu). The reanalysis product is based on the coupled physical-biogeochemical model system NEMO-SCOBI (Nucleus for European Modelling of the Ocean – Swedish Coastal and Ocean Biogeochemical model). The data were extracted for a water depth of 1.5 m. Annual average salinity and summer (June, July and August) average temperature were calculated for each station for the year of sampling, 2017.

### Data analysis

Prevalence, expressed as the percentage of hosts infected with one or more individuals of a particular parasite species, was calculated for each parasite species found in either periwinkles or mussels (see online Resource Fig. S1). Parasite mean intensity (the number of a particular species of parasite among the infected members of a particular host species) and mean abundance (the number of parasites among all members of a particular host species) were calculated for the parasites in mussels in each sampling station (online Resource Table S1). Species richness was also calculated for each sampling station (online Resource Fig. S1). Intensities and abundance of non-trematode parasites/symbionts were not considered since numbers were extremely low. For plots and analyses, densities of periwinkle populations were calculated as means of the extreme values for each density class (i.e. 0–30, 30–50, 50–100). Then, the density of infected periwinkles was obtained by multiplying the prevalence of the considered trematode species by the periwinkle population density.

All statistical analyses were performed using software R 3.5.0 (R Core Team, 2018). Differences in the prevalence of trematodes in periwinkles and mussels between sampling locations were tested using Fishers' exact test. The effects of salinity, host size (defined as shell length of mussels, measured with a calliper) and density of infected periwinkles on the prevalence of *R. roscovita* and *H. elongata* in mussels (response variables) were tested using generalized linear mixed models (GLMMs) with binomial distribution and logit link function. In all models, prevalence was considered as parasite species presence/absence per individual mussel. The effects of the same predictors on the abundance of *R. roscovita* and *H. elongata* were tested using zero inflated GLMMs with a negative binomial distribution (package glmmTMB). A zero-inflated alternative was preferred over a standard GLMM, since absences represented a large proportion of the count data, especially for the lower salinity stations. The zero-inflated extensions indeed more appropriately modelled the excess of zeros and overdispersion of the data than the Poisson and negative binomial GLMM that were initially fitted. In all models, the identity of stations was included as random factor. Similar to prevalence, abundance was considered at the individual level, as the number of metacercariae of *R. roscovita* or *H. elongata* in infected and non-infected individual hosts. Temperature was not included in the models as the range of average summer temperature among stations in 2017 was too small to detect potential effects (between 15.7 and 17.5°C).

Before running the models, collinearity among predictors was examined through the analysis of bivariate scatter plots and Pearson correlations (online Resource Figs S2 and S3). Moreover, we used the variance inflation factor (VIF) to further test for collinearity in the fitted GLMM models with binomial distribution (packages ‘mctest’) (online Resource Table S3). Computing VIFs was not possible for the zero-inflated models as VIFs appear not sensible in these types of models. For prevalence, GLMMs derived from all potential combinations of included predictors (i.e. salinity, mussel host size and density of infected periwinkles) were automatically constructed using the ‘dredge’ function (package MuMIn). All models were compared using the Akaike information criterion corrected for small sample size (AICc), delta AICc (ΔAICc) and the AICc weights (AICcw) (online Resource Table S2). The models with the lowest AICc were chosen as the best models. The same procedure was applied for the zero-inflated models for abundance.

## Results

### Parasite infections in periwinkles and mussels

Both periwinkles and mussels were found along the salinity gradient from 13 to 22 from Niendorf to Hou (Table 1). The two most common trematode species infesting periwinkles and mussels as intermediate host, *R. roscovita* and *H. elongata*, had a mean prevalence of 6.1 and 9.7% in periwinkles, and 41.7 and 52.7% in mussels, respectively (Table 2). Other parasites found in periwinkles were *Cryptocotyle lingua*, *Microphallus pygmaeus* and *Podocotyle atomon* (Table 2; online Resource Fig. S1A). Ciliates were also found, with lower prevalence (Table 2; online Resource Fig. S1A). Other parasite species found in mussels were turbellaria (*P. cardii*), nematodes, copepods and ciliates (Table 2; online Resource Fig. S1C). Mean species richness of parasites in individual periwinkles varied from 1.0 to 1.4 between locations (online Resource Fig. S1B). In mussels, the mean species richness in individual mussels varied from 1.0 to 2.2 (online Resource Fig. S1D). For periwinkles, the highest mean species richness of parasites in individual periwinkles was found in Årøsund (salinity: 18.5; mean species richness: 1.4). For mussels, the highest mean species richness in individual hosts was found in Laboe (salinity: 16.5; mean species richness: 2.2).

**Table 2.** List of parasite and symbiont/commensal species identified in *Littorina littorea* and *Mytilus edulis* as first and second intermediate hosts, respectively, their taxon, their mean prevalence among sampling stations (pooled over stations) ± standard error (s.e.), as well as all other hosts known to be part of the parasites' life cycle

Host species	Parasite species	Taxon	Mean prevalence ± s.e.	First intermediate host	Mean intensity	Mean abundance	Second intermediate host	Final host
*L. littorea*	*R. roscovita*	Platyhelminthes	6.1 ± 4.2	*L. littorea*	–	–	Molluscs	Sea birds
	*Himasthla elongata*	Platyhelminthes	9.7 ± 6.0	*L. littorea*	–	–	Molluscs	Sea birds
	*Cryptocotyle lingua*	Platyhelminthes	6.2 ± 2.6	*L. littorea*	*–*	*–*	Fishes	Sea birds
	*Microphallus pygmaeus*	Platyhelminthes	1.8 ± 1.2	*L. littorea*	–	–	*L. littorea*	Sea birds
	*Podocotyle atomon*	Platyhelminthes	1.6 ± 0.7	*L. littorea*	–	–	Amphipods	Fishes
	*Tricodina* sp.	Ciliophora	0.6 ± 0.6	–	–	–	–	–
*M. edulis*	*R. roscovita*	Platyhelminthes	41.7 ± 17.0	*L. littorea*	123	90.0	Molluscs	Sea birds
	*H. elongata*	Platyhelminthes	52.7 ± 15.2	*L. littorea*	38	22.5	Molluscs	Sea birds
	*Paravortex cardii*	Platyhelminthes	19 ± 9.5		3	0.6		
	*unknown trematode*		1.3 ± 1.3	–	154	1.5	Molluscs	–
	*Modiolicola* sp.	Arthropoda	0.5 ± 0.5	–	–	–	–	–
	*Trichodina* sp.	Ciliophora	4.2 ± 2.2	–	–	–	–	–
	*Nematodes*	Nematoda	1.1 ± 1.1	–	–	–	–	–

**Fig. 2.**
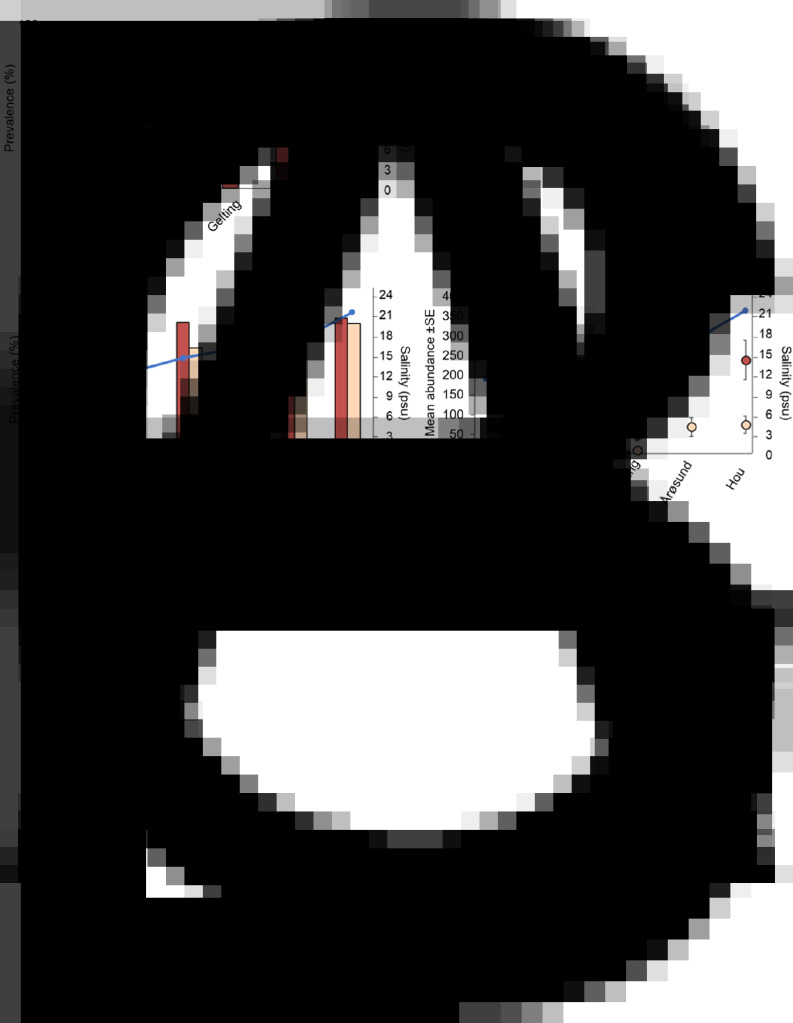
(Colour online) (A) *Renicola roscovita* and *Himasthla elongata* prevalence in *Littorina littorea*, and (B) *Renicola roscovita* and *Himasthla elongata* prevalence and (C) mean abundance in *Mytilus edulis* and mean annual salinity (indicated by the black dots and the line). Whiskers in (C) represent standard error (s.e.).

Spatially, in both, periwinkles and mussels, *R. roscovita* and *H. elongata* prevalences were highest in Laboe, Gelting, Årøsund and Hou (salinity ranging from 16.5 to 22.2; Figs 2A and B). Specifically, in mussels *R. roscovita* and *H. elongata* prevalences were significantly higher in Hou, Årøsund, Gelting and Laboe than in Niendorf and Großenbrode (*P* < 0.001). In periwinkles, *R. roscovita* and *H. elongata* prevalences were higher in Årøsund (*P* < 0.001) than in other stations. The highest abundances of *R. roscovita* and *H. elongata* in *M. edulis* were detected in Årøsund and in Hou (Fig. 2C).

### Effects on parasite prevalence in mussels

The model including only salinity and mussel size was identified as the most parsimonious model for explaining *R. roscovita* prevalence in mussels. This model was 2.3 times more likely (based on the AICcw ratio) than the second most parsimonious one, which also included the density of infected periwinkles (online Resource Table S2). Prevalence of *R. roscovita* in mussels increased significantly with salinity (Table 3) and mussel size (Table 3). Density of infected periwinkles and *R. roscovita* prevalence in mussels were unrelated. The two predictors included in the model explained 50% of the variance in *R. roscovita* prevalence. The model including just salinity was identified as the most parsimonious model for explaining *H. elongata* prevalence. This model was 2.10 times more likely than the second most parsimonious model, which also include density of infected periwinkles (online Resource Table S2). *Himasthla elongata* prevalence in mussels significantly increased with salinity (Table 3). Salinity explained 44% of the variance in *H. elongata* prevalence.

**Table 3.** GLMMs following binomial distribution on the effects of density of infected periwinkles and mussel size on the prevalence of *R. roscovita* and *H. elongata* in blue mussels (*M. edulis*)

Trematode species	Predictors	Estimate	s.e.	*Z* value	*P* value
*R. roscovita*	Intercept	−17.500	5.295	−3.305	<0.001***
	Salinity	0.749	0.285	2.624	0.008**
	Size	0.102	0.034	3.020	0.002**
*H. elongata*	Intercept	−11.515	3.166	−3.637	<0.001***
	Salinity	0.672	0.180	3.726	<0.001***

The symbols ‘*’, ‘**’ and ‘***’ indicate *P* values <0.05, <0.01 and <0.001, respectively. s.e.: standard error.

### Effects on parasite abundance in mussels

The model including all predictors was identified as the most parsimonious model for explaining *R. roscovita* abundance in mussels. This model was 2.09 times more likely than the second most parsimonious model (online Resource Table S2). *Renicola roscovita* abundance increased with salinity, density of infected periwinkles and mussel size (Table 4).

**Table 4.** Zero–inflated models with a negative binomial distribution on the effects of salinity, mussel size (length) and density of infected periwinkles on the abundance of *R. roscovita* and *H. elongata* in mussels (*M. edulis*)

Parasite species	Predictors	Estimate	s.e.	*Z* value	*P* value
*R. roscovita*	Intercept	0.2344	1.3779	0.170	0.8648
	Salinity	0.1542	0.0658	2.345	0.0190*
	Size	0.0318	0.0112	2.819	0.0048**
	Density of infected periwinkles	0.0518	0.0179	2.894	0.0038**
*H. elongata*	Intercept	−0.4853	0.9789	−0.496	0.6200
	Salinity	0.2010	0.0502	4.001	<0.001***
	Density of infected periwinkles	0.0282	0.0102	2.755	0.0058**

The symbols ‘*’, ‘**’ and ‘***’ indicate *P* values <0.05, <0.01 and <0.001, respectively. s.e.: standard error.

The model including only salinity and density of infected periwinkles was identified as the most parsimonious model for explaining *H. elongata* abundance. The AICc was 2.76 more likely than the second most parsimonious model including mussel size (online Resource Table S2). *Himasthla elongata* abundance increased with salinity and density of infected periwinkles (Table 4).

## Discussion

### General patterns of parasite prevalence and intensity

The parasite community in periwinkles (*L. littorea*) and mussels (*M. edulis*) appeared to be dominated by trematodes, among which the species *R. roscovita* and *H. elongata* showed the highest prevalence in both host species. In the periwinkle, *C. lingua* was the third species with high prevalence. These patterns of prevalence were also observed by Lauckner (1984), who, however, reported *C. lingua* prevalence to be slightly higher than that of *R. roscovita* and *H. elongata* in periwinkles collected in shallow waters. We found *C. lingua* and *M. pygmaeus* generally more prevalent at salinities below 17.8, whereas *R. roscovita* and *H. elongata* were dominant at salinities above 17.8. Our findings are coherent with other previous studies from the Baltic Sea (Reimer, 1970; Möller, 1978; Lauckner, 1984), revealing a dominance of *C. lingua* and *M. pygmaeus* in brackish waters. In the case of *M. pygmaeus*, this success could derive from a shorter life cycle, involving one single intermediate host only, where cercariae directly develop into metacercariae, and are likely less subjected to environmental stress compared to free-living cercariae (Kesting *et al*., 1996; Marcogliese, 2001). In the case of *C. lingua*, the higher spread might be attributed to the motile second intermediate host (i.e. fish) and to the protection from osmotic damage provided by the fish intestine (Möller, 1978).

### Role of biotic factors

The abundance of *R. roscovita* and *H. elongata* in mussels increased with the density of infected periwinkles. These results are generally coherent with the findings of Thieltges and Reise (2007) from the Wadden Sea (North Sea), who noted a positive effect of first intermediate host density and host size on prevalence and abundance of common trematode species in cockle second intermediate hosts. An increase in abundance at higher infected first intermediate host density probably results from an increased supply of infective cercarial stages released from first intermediate hosts, which in turn leads to increased infection levels in the mussel hosts as infection levels in mussels are known to be dose-dependent (Liddell *et al*., 2017).

Prevalence and abundance of *R. roscovita* in mussels increased with host size (mussel length), whereas this was not observed for *H. elongata*. A positive correlation between parasite prevalence, abundance and host size was already shown in previous studies in the North Sea (Thieltges *et al*., 2010; Goedknegt, 2019) and could be attributed to higher filtration rates occurring in larger mussels (Nikolaev *et al*., 2006), which enhance the chance of more cercariae to enter the mussel through the inhalant current (Wegeberg *et al*., 1999; Thieltges and Reise, 2007). In terms of time of exposure, the age of mussels could be another explanation and the higher number of metacercariae detected in larger – and older – mussels could be a result of parasite accumulation through time (Nikolaev *et al*., 2006). At this stage, we can only speculate why this did not occur for *H. elongata*. One possibility is that contrarily to *H. elongata*, *R. roscovita* metacercariae are known to grow inside the second intermediate host (Lauckner, 1980, 1983). This characteristic may affect *R. roscovita* cercariae preference for larger-size host individuals. Alternatively, *R. roscovita* cercariae are mainly transmitted during filtration of the host through the inhalant current. A higher filtration rate could therefore be more relevant for *R. roscovita* than *H. elongata* cercariae, which also transmit through active penetration of the hosts' foot.

Although the density and traits of the infected intermediate host are certainly affecting parasite transmission, other factors such as cercarial longevity or behaviour are relevant for transmission success. Previous studies have shown that the longevity of *R. roscovita* can reach 30 h and *Himasthla* sp. may even survive for 48 h at 20°C (Thieltges and Rick, 2006; de Montaudouin *et al*., 2016). Considering that cercarial functional longevity (time until when cercariae are no longer able to infect) represents between 20 and 50% of the survival time, the potential of infection success of the two species might differ. Also, parasite species exhibit different cercarial emergence and swimming behaviours. Renicolidae are known to produce numerous cercariae showing broad dispersion along the water column in the first hours of emergence, which start sinking after 6 h of life. Contrarily, *H. elongata* produces fewer cercariae swimming constantly to the bottom, showing positive geotactic response and limited dispersal ability (Nikolaev *et al*., 2017). These two different strategies suggest that the vertical distribution of the intermediate hosts may impact cercariae transmission and therefore metacercariae abundance. However, our study was carried out in extremely shallow waters (<50 cm depth), where periwinkles and mussels are overlap in their zonation, which makes the effect of vertical distribution, in our case, negligible.

### Role of salinity

*Renicola roscovita* and *H. elongata* prevalence and abundance in mussel hosts, significantly increased with increasing salinity within the range investigated here. Besides a general positive correlation among parasite prevalence and salinity, we found a slight difference between the two main parasite species, *R. roscovita* and *H. elongata*. Indeed, the prevalence and abundance of *H. elongata* correlated more strongly with salinity than that of *R. roscovita*. As reported by Lauckner (1984), *H. elongata* infects periwinkles through swimming and salinity-sensible miracidia. In *R. roscovita*, eggs are directly ingested by *L. littorea*, and consequently possibly less impacted by salinity changes.

There is no other field study available that correlates parasite prevalence and abundances in intermediate hosts of trematodes to the steep salinity gradient within the western Baltic Sea. Along a salinity gradient from 4 to 30, in their sampling along the Ria de Aveiro coast, Magãlhaes *et al*. (2018) did not observe salinity effects on parasite abundance in cockles (*Cerastoderma edule*). However, the high spatial homogeneity detected was probably caused by the low abundance of parasites in general (Magãlhaes *et al*., 2018). This is not the case for the western Baltic Sea coast, where high abundances of trematodes were previously reported (Werding, 1969; Lauckner, 1984; Zander, 1998). Also, Goedknegt *et al*. (2019), during their sampling in the Wadden Sea, did not detect any significant correlation between prevalence and abundance of *H. elongata* and *R. roscovita* and salinity. However, their study was conducted in a different system, where the salinity gradient goes from 22 to 31, the lowest limit corresponding to our highest values. Other field studies focusing on parasite diversity, but not on prevalence and abundance, identified salinity as one of the most critical factors influencing parasite distribution, superimposing even on local effects such as eutrophication (Schmidt *et al*., 2003; Blanar *et al*., 2011; Poulin *et al*., 2011).

As previously observed by Zander (1998), trematode distribution could be an indirect consequence of the intermediate host (periwinkles and mussels) distribution, which themselves have limits of adaptive osmoregulation to changes in salinity. A critical threshold for periwinkle activity is represented by salinities lower than 13 psu (Taylor and Andrews, 1988). Mussels of the genus *Mytilus* spp. instead are still found in the Northern Baltic Sea at salinities below 6.5, where they reach their margin of tolerance (Riisgård *et al*., 2013). However, *Mytilus* populations of the Baltic Sea show a clear pattern of interspecific gene flow among *M. edulis* and *Mytilus trossulus*. In the central Baltic Sea, the genotype is *M. trossulus*-like, instead in the western Baltic Sea, at salinity around 15 (Kiel Fjord, including Laboe) the genotype is *M. edulis*-like (Stuckas *et al*., 2017). Salinity effects can also strongly impact the free-living stages of trematodes. Generally, if we assume the marine nature of the trematode species analysed, their distribution and reproductive capacity are directly affected by the hydrology dynamics (i.e. shifts in salinity) of the habitat, as mentioned by Schmidt *et al*. (2003). This tight association among marine trematodes and habitat hydrology is confirmed by experimental studies, which detected cercarial emergence, survival and transmission negatively affected by lower salinities (Lei and Poulin, 2011; Studer and Poulin, 2012; Bommarito *et al*., 2020). Negative effects of reduced salinity in cercarial emergence might be attributed to a stress response by the first intermediate host (Lei and Poulin, 2011; Bommarito *et al*., 2020), whereas negative effects on survival and infectivity could be attributed to the vulnerability of the cercaria itself (Pietrock and Marcogliese, 2003). In their experimental approach, Bommarito *et al*. (2020) simulated a salinity range of 13–19. The study found *H. elongata* emergence, activity and infectivity to be significantly reduced at low salinity (i.e. 13), probably due to osmotic stress in cercariae. In the same study, not only infectivity but also mussel susceptibility to infection significantly diminished with decreasing salinity, highlighting the mediating role of the host during the infection.

### Comparison of experimental and field studies

The cumulative experimental results detected in previous studies (Studer and Poulin, 2012; Bommarito *et al*., 2020) are matching with our field data, generally suggesting that at reduced salinity trematode transmission to the second intermediate host drastically decreases due to the combined osmotic stress experienced by both, the cercariae and their hosts. However, in the field the effect of salinity on transmission might be further enhanced or buffered by additional biotic components. Our field results detected a positive effect of first intermediate host density in *H. elongata* abundance, and as first intermediate host density clearly increased at the highest salinities (Table 1), this further promotes the chance of trematode transmission. In the future, salinity in the Baltic Sea is expected to decrease (by around 2 psu by 2100, see Meier *et al*., 2012). With a salinity around 13 representing the margin of tolerance of *L. littorea* (Hylleberg and Christensen, 1978; Taylor and Andrews, 1988), locations which currently present an average salinity of 16 will possibly be subjected to a decrease in periwinkle host population density, which may lead to a decrease in abundance of *H. elongata*.

The other biotic factor which positively affected trematode prevalence and abundance (in *R. roscovita*) was mussel size. The range of mussel sizes used by Bommarito *et al*. (2020) was similar to that observed in this field study (mussels of 40–50 mm). Average mussel size in our field study appeared to increase with salinity, from 40 (13.8) to 47–49 (17.8–22). This suggests that at higher salinities cercarial infectivity and host susceptibility might further raise due to cercariae preference for larger mussels and an increase in mussel filtration rate. With Baltic Sea freshening, a reduction in mussel size might also occur and this phenomenon has already been observed in the dwarfed mussels (hybrids between *M. trossulus* and *M. edulis*) of the proper Baltic Sea (Riisgård *et al*., 2013). At salinities below 16 the negative effect of freshening might thus be exacerbated by smaller mussel size. However, to confirm this hypothesis, the additive effects of salinity and downstream host size will need to be experimentally tested in different marine trematode species.

Finally, it is important to stress that the transmission success depends also on the genotypes of both parasite and host. Osnas and Lively (2005), working in freshwater systems of New Zealand, showed that sympatric trematodes induced similar immune response to host snails as allopatric ones, but the response of the host against sympatric parasites was less effective. Nevertheless, this is not a general rule, as allopatric hosts can sometime be less resistant compared to sympatric ones (Kaltz and Shykoff, 1998). Yet, it remains unclear whether local adaptation plays a role in our parasite–host system. As both the snail hosts (via pelagic larvae) and the parasites (via their bird definitive hosts) show high dispersal and connectivity capability, local adaptation on the scale of our study area may only play a minor role. Future research should investigate the potential role of coevolutionary processes along the salinity gradient of the Baltic Sea.

## Conclusions

Our study emphasized the importance of salinity, first intermediate host density and mussel size as drivers of trematode infection levels in mussels of the Baltic Sea. Under global change, the increasing osmotic stress in a freshening Baltic Sea will affect the distribution of the first intermediate periwinkle hosts as well as the size of mussels, possibly leading to a reduction of infection levels in mussels. Furthermore, the correlation of trematode prevalence and abundance with salinity observed in our study aligns well with experimental data obtained for the same host–parasite system, and both approaches suggest that the freshening of the Baltic Sea could lead to a reduction of trematode transmission success.

## Supplementary material

For supplementary material accompanying this paper visit https://doi.org/10.1017/S0031182020002188. click here to view supplementary material
