# Supplementary material for: Effects of first intermediate host density, host size and salinity on trematode infections in mussels of the south-western Baltic Sea
Source: Parasitology. 2020 Nov 20;148(4):486–94. doi: 10.1017/S0031182020002188 (PMC7938341; doi:10.1017/S0031182020002188)
Supplement: Supplementary file 1 [file S0031182020002188sup001.pdf]

Electronic Supplementary Material

Title: Effects of upstream host density, host size and salinity on parasite infections in mussels the south-western Baltic Sea

List of authors: Claudia Bommarito [1,2], David W Thieltges[3], Christian Pansch [4], Francisco R. Barboza [1], Fabio Pranovi [2], Martin Wahl [1]

Affiliations:

[1] GEOMAR Helmholtz Centre for Ocean Research Kiel, Department of Marine Ecology; 24105, Kiel, Germany

[1] University Ca' Foscari of Venice, Department of Environmental Sciences, Informatics and Statistics; 30172, Venice, Italy

[3] NIOZ Royal Netherlands Institute for Sea Research, Department of Coastal Systems, and Utrecht University, P.O. Box 59, 1790, AB Den Burg Texel, The Netherlands

[4] Environmental and Marine Biology, Åbo Akademi University, Artillerigatan 6, 20520 Åbo, Finland

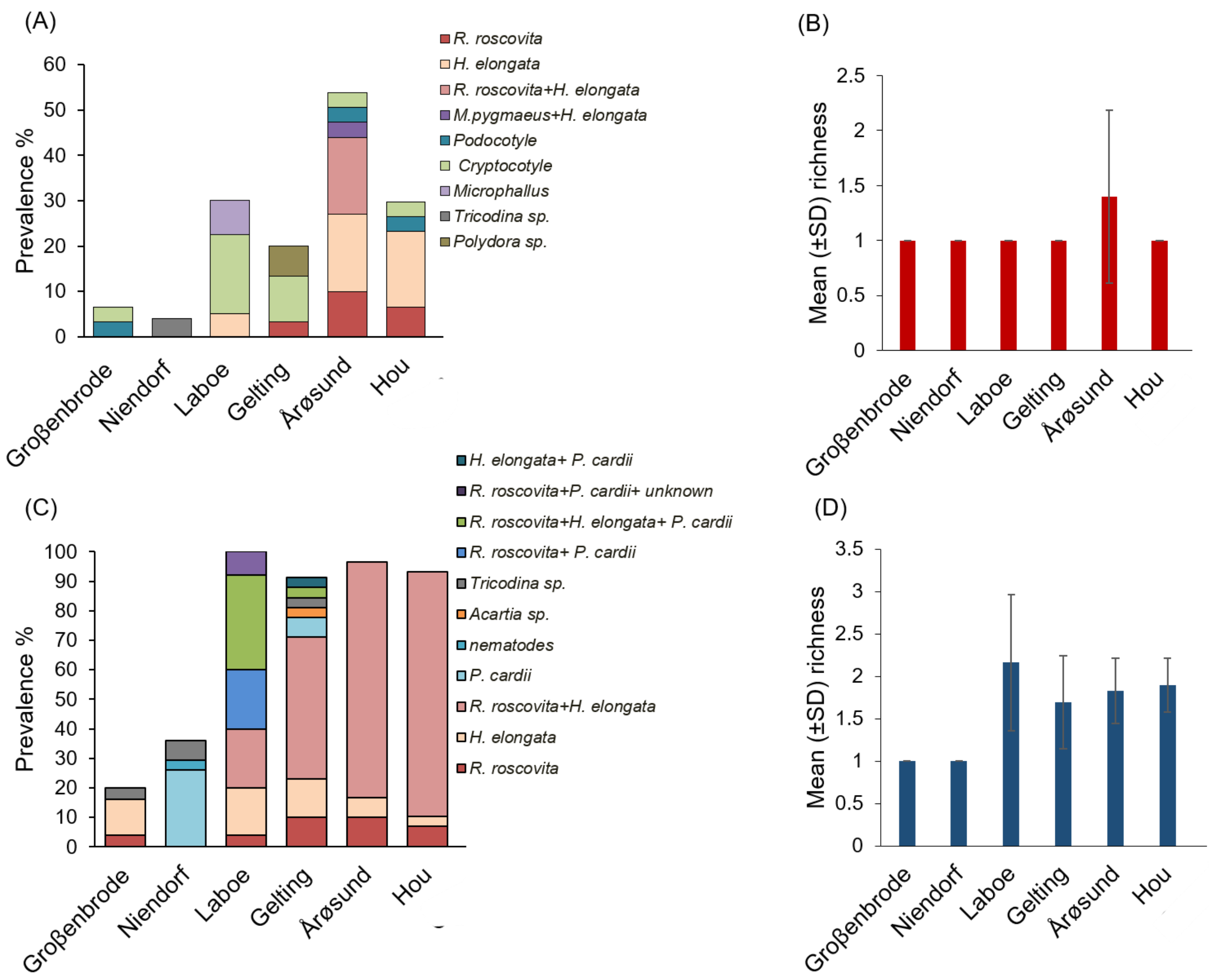

Fig.S1 (**A**) Parasite prevalence and (**B**) mean species richness of parasites in individuals of *Littorina littorea*, and (**C**) prevalence and (**D**) mean richness of parasites in individuals of *Mytilus edulis*, in each sampling station. All sampling locations are ordered from low (Grossenbrode) to high salinity (Hou; see also Table 1) conditions, considering the annual mean salinities registered in 2017. Prevalence of co-infections are shown separately. Whiskers represent standard deviations (SD).

Table S1. Spatial pattern of parasites found in *Mytilus edulis*. For each sampling station, prevalence (%, P), mean intensity (I) (±SE), mean abundance (A) (±SE) of each parasite species are presented.

Station	*Renicola roscovita*			*Himasthla elongata*			*Paravortex cardii*			*Unknown trematode*			*Modiolicola sp.*			*Tricodina* sp.			*Nematodes*		
	P	I	A	P	I	A	P	I	A	P	I	A	P	I	A	P	I	A	P	I	A
Hou	86	276.4±56	238.3±50	83	86.2±56	74.3±21	0	0	0	0	0	0	0	/	0	0	0	0	0	0	0
Årøsund	90	321.8±57	289.6±54	86	78.8±57	68.3±24	0	0	0	0	0	0	0	/	0	0	0	0	0	0	0
Gelting	60	73.6±20	48.2±14	66	12.2±20	8.4±3.1	13	1.7±0.7	0.2±0.1	0	300±0	10.3±10.1	3	1±0	0.03±0	3	0	0	0	0	0
Laboe	84	61±13	53.8±12	68	9.4±13	6.4±2.4	60	5.3±1.7	3.3±1	8	8±0	0.6±0.5	0	0	0	0	0	0	0	0	0
Niendorf	0	0	0	0	0	0	33	2±0	0.6±0.2	0	0	0	0	0	0	10	0	0	6.6	1	0.06±0
Groβenbrode	4	1±0	0.04±0.04	12	1.0±0	0.12	8	1.9±0.6	0.1±0.1	0	0	0	0	0	0	12	0	0	0	0	0

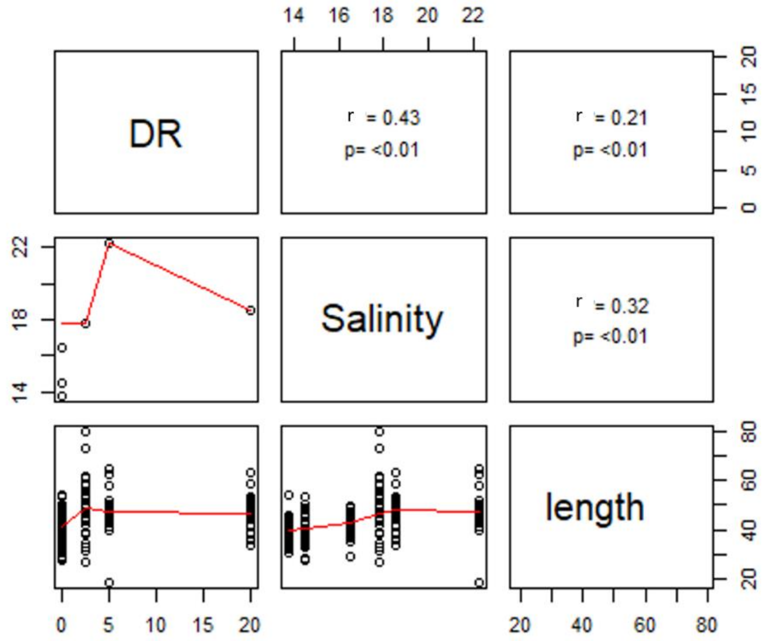

Fig.S2 Pairplot for all putative environmental drivers contributing to prevalence and abundance of *Renicola roscovita*, where "DR" is density of periwinkles infected with *Renicola roscovita* and "length" is mussel length. The lower diagonal elements contain the correlations.

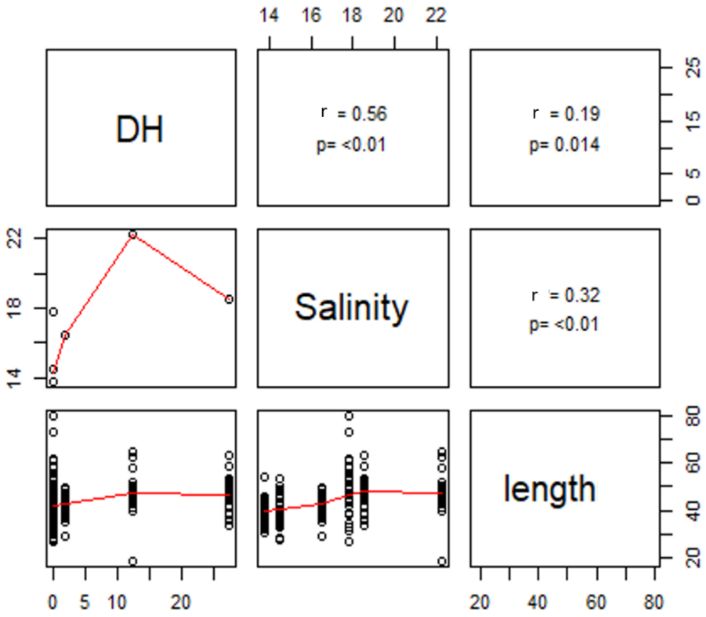

Fig.S3 Pairplot for all putative environmental drivers contributing to prevalence and abundance of *Himasthla elongata*. where "DH" is density of periwinkles infected with *Himasthla elongata* and "length" is mussel length. The lower diagonal elements contain the correlations.

Table S2. Model selection for the generalised linear mixed models (GLMMs) and zero-inflated GLMMs applied for *Renicola roscovita* and *Himasthla elongata* prevalence and abundance, respectively. Predictors included in the model are indicated with "+" and predictors excluded with "-". The degrees of freedom (df), the Akaike Information Criterion corrected for small sample size (AICc), the delta AICc (ΔAICc) and AICc weights (AICcw) are presented for each model. The model with the lowest AICc is presented in bold.

Dependent Variable	Predictors						
	Salinity	Mussel size	Density of infected periwinkles	df	AICc	ΔAICc	AICcw
Prevalence of *R. roscovita*	+	+	+	5	120.4	1.64	0.232
	**+**	**+**	**-**	**4**	**118.7**	**0.00**	**0.534**
	+	-	+	4	129.2	10.48	0.003
	-	+	+	4	122.2	3.38	0.098
	+	-	-	3	127.8	8.97	0.006
	-	+	-	3	121.7	2.96	0.122
	-	-	+	3	131.5	12.75	0.001
	-	-	-	2	131.3	12.49	0.001
Prevalence of *H. elongata*	+	+	+	5	153.7	3.06	0.089
	+	+	-	4	152.2	1.56	0.189
	+	-	+	4	152.2	1.49	0.196
	-	+	+	4	156.6	5.95	0.021
	+	-	-	3	**150.6**	**0.00**	**0.413**
	-	+	-	3	157.2	6.58	0.015
	-	-	+	3	155.2	4.49	0.044
	-	-	-	2	155.8	5.13	0.032
Abundance of *R. roscovita*	+	+	+	8	**1254.7**	**0.00**	**0.421**
		+	+		1256.2	1.48	0.201
	+	+	-		1256.5	1.80	0.171
	-	+	-		1257.5	2.78	0.105
	+	-	+		1258.4	3.67	0.067
	-	-	+		1263.0	8.26	0.007
	+	-	-		1263.0	8.30	0.007
	-	-	-		1264.7	10.00	0.003
Abundance of *H. elongata*	+	-	+	7	**960.7**	**0.00**	**0.636**
	+	+	-	8	962.8	2.04	0.230
	+	-	-	6	**965.1**	**4.32**	**0.074**
	+	+	-	7	967.2	6.45	0.025
	-	-	+	6	968.9	8.18	0.011
	-	+	+	7	970.3	9.57	0.005
	-	-	-	5	971.5	10.79	0.003
	-	-	-	6	973.2	12.43	0.001

Table S3 VIF values of the Generalised linear mixed models (GLMMs) following binomial distributions applied for *Renicola roscovita* and *Himasthla elongata* prevalence and including all the fixed factors (Salinity, density of infected periwinkles and mussel size (length).

**Predictors**	***Renicola roscovita***	***Himasthla elongata***
Salinity	1.417895	1.178867
Density of infected periwinkles	1.400152	1.000061
Mussel size	1.018318	1.178802

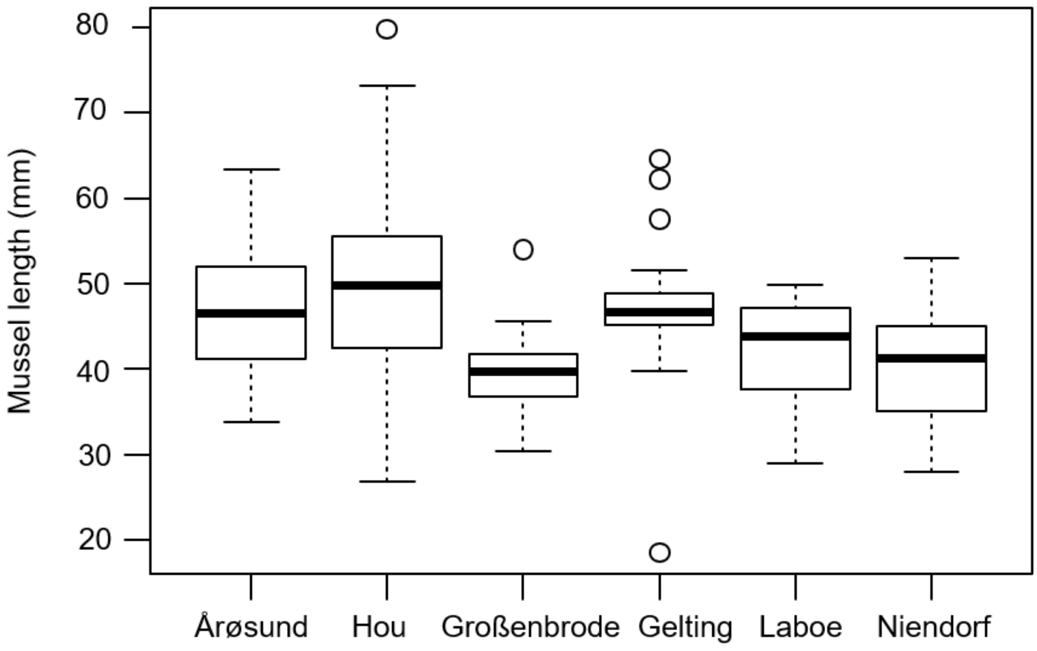

Fig. S4 Mussels lengths at the different sampling stations. The boxes represent the 75 (upper) and 25 (lower) percentile, the whiskers denote the lowest and the highest value, the black lines within the boxes the medians and the black dots the outliers.
